# Functional Insights into Protein Kinase A (PKA) Signaling from *C. elegans*

**DOI:** 10.3390/life12111878

**Published:** 2022-11-14

**Authors:** Fereshteh Sadeghian, Perla G. Castaneda, Mustafi R. Amin, Erin J. Cram

**Affiliations:** 1 Department of Bioengineering, Northeastern University, Boston, MA 02115, USA; 2 Department of Biology, Northeastern University, Boston, MA 02115, USA

**Keywords:** PKA, cAMP, calcium signaling, *C. elegans*, physiology, behavior

## Abstract

Protein kinase A (PKA), which regulates a diverse set of biological functions downstream of cyclic AMP (cAMP), is a tetramer consisting of two catalytic subunits (PKA-C) and two regulatory subunits (PKA-R). When cAMP binds the PKA-R subunits, the PKA-C subunits are released and interact with downstream effectors. In *Caenorhabditis elegans* (*C. elegans*), PKA-C and PKA-R are encoded by *kin-1* and *kin-2,* respectively. This review focuses on the contributions of work in *C. elegans* to our understanding of the many roles of PKA, including contractility and oocyte maturation in the reproductive system, lipid metabolism, physiology, mitochondrial function and lifespan, and a wide variety of behaviors. *C. elegans* provides a powerful genetic platform for understanding how this kinase can regulate an astounding variety of physiological responses.

## 1. Introduction

Protein kinase A (PKA) is a 3′-5′-cyclic adenosine monophosphate (cAMP)-dependent kinase that is at the center of diverse biological functions in numerous systems. Inactive PKA is composed of two catalytic (PKA-C) and two regulatory (PKA-R) subunits. The binding of cAMP to the PKA-R subunits activates the enzyme, causing the release of PKA-C. The PKA-R subunits, therefore, inhibit PKA-C in the absence of cAMP. Once released, PKA-C interacts with multiple downstream effectors and regulates lipid metabolism [1], cell migration [2], and vasodilation [3], among many other functions [4]. While numerous genes encode the regulatory and catalytic subunits of PKA in mammals, complicating the study of PKA, *Caenorhabditis elegans* (*C. elegans*) only has a single gene encoding the regulatory subunit of PKA, *kin-2*, and one gene encoding the catalytic subunit, *kin-1.* This review focuses on the contribution *C. elegans* has made to our understanding of the function and biological role of PKA.

## 2. Isoforms

Humans have three genes encoding PKA-C: PRKACA, PRKACB, and PRKACG, and two types of PKA-R’s: type PKA-RI (PKA-Riα and PKA-Riβ) and type PKA-RII (PKA-RIIα and PKA-RIIβ). Holoenzyme expression and distribution is largely determined by the type of regulatory subunit [5,6,7]. The PKA-RIIβ subunit is found in endocrine, brain, fat, and reproductive tissues. The PKA-RIα and PKA-RIIα subunits are expressed ubiquitously [5,6] and PKA-RIIβ is enriched in the mitochondria. PKA-C can undergo post- and co-translational modifications [8], including myristoylation of the N-terminus, which increases PKA-C membrane affinity [9]. PKA-RIα has the highest binding affinity, followed by PKA-RIIα and PKA-RIIβ [5,6].

In *C. elegans*, PKA is encoded by one catalytic subunit, KIN-1/PKA-C, and one regulatory subunit, KIN-2/PKA-R [10]. The PKA binding sites on *kin-2* are not very well conserved [11]. With only one PKA-C subunit for PKA-R to coordinate with, rather than multiple forms encoded by different loci, the two subunits may have co-evolved; changes in the PKA-R locus could be compensated for by sequence changes in the PKA-C locus [11]. KIN-1/PKA-C is 82% identical to the mammalian catalytic subunit of PKA, and KIN-2 is most closely related to type I mammalian PKA regulatory subunit [12]. Proteomic analysis identified 419 potential PKA substrates with 630 potential PKA binding sites in *C. elegans* [11].

In *C. elegans*, expression of specific isoforms of the catalytic subunit *kin-1* could contribute to the specificity of PKA activity [13,14]. The *kin-1* gene has a total of 13 exons, including six 5′ exons (N′1–N′6), and at least 12 different *kin-1* isoforms are expressed [14]. Depletion of N′3 *kin-1* isoform led to paralysis and egg-laying defects, while knockdown N′4 variants resulted in no apparent phenotypes [15]. Isoforms containing N′3 and N′4 are not targets of myristoylation [14], while the N′1 isoforms are N-myristoylated. This protein modification may prohibit docking of the N-terminal domain to a hydrophobic pocket in PKA-C, possibly affecting intracellular targeting and differentially regulating *kin-1* function [16]. Although one gene, *kin-2*, encodes PKA-R in *C. elegans*, diversity of function might be achieved through differential expression of its three isoforms. Some *kin-2* isoforms lack the typical dimerization/docking domains, implying they do not form the tetrameric PKA holoenzyme or interact with AKAP proteins, and suggesting these PKA-R isoforms might have other, unknown, functions [17].

## 3. AKAPs

**Figure 1:**
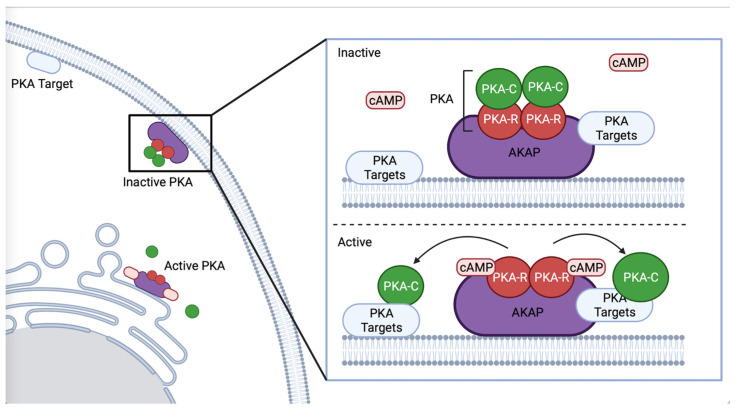
**Schematic representation of AKAP binding PKA**. AKAP binds the regulatory subunit of PKA, regulating PKA’s subcellular localization and co-locating PKA with specific phosphorylation targets, such as proximal to the ER or plasma membrane. Created with BioRender.com (accessed on 31 October 2022).

PKA participates in many signaling pathways. A kinase anchoring proteins (AKAP) scaffold PKA and regulate signaling output by enabling association with specific effectors (Figure 1) [4,18]. Regulatory subunits are bound by AKAP until PKA is activated, allowing for spatial and temporal control of PKA [7,19,20]. AKAP-1 is the best characterized AKAP in *C. elegans* [21], and has a primarily neuronal expression pattern [22]. AKAP-1 has a high affinity for KIN-2/PKA-RI [21]. Although *C. elegans* expresses only one AKAP, other proteins, such as ERM-1, an ortholog of ezrin, which acts as an AKAP in mouse gastric parietal cells [23], may also function as an AKAP to PKA in *C. elegans*. ERM-1 is expressed in epithelial tissues such as the intestine and spermatheca, where it regulates apical polarization, junction formation [24], cortical actin organization, and lumen formation [25], processes in which PKA could play a role.

## 4. Activation of PKA by G-Proteins

**Figure 2:**
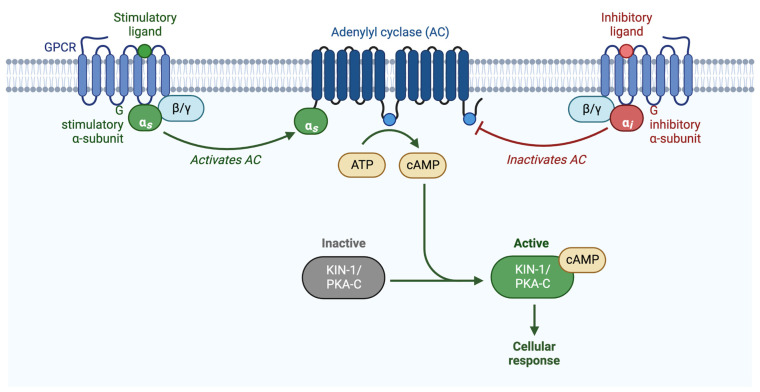
**Schematic representation of G-protein activation of PKA**. PKA is activated when the regulatory subunit binds to cAMP, releasing the catalytic subunit. cAMP is produced by adenylyl cyclase, which is either activated by Gαs, or inhibited by Gαi/o. Adapted from “Activation of Protein Kinase A (PKA)”, by BioRender.com (2022). Retrieved from https://app.biorender.com/biorender-templates (accessed on 27 October 2022).

The second messenger, cyclic AMAP (cAMP), is produced by adenylyl cyclase, which converts adenosine triphosphate (ATP) to cAMP. PKA is activated by the binding of cAMP to the PKA-R subunits, which releases PKA-C. cAMP levels are reduced by phosphodiesterases (PDEs), which convert cAMP into AMP [26]. Adenylyl cyclases are commonly regulated by G-protein signaling. Heterotrimeric G-proteins consist of an α and a βγ subunit, which, when activated by an upstream G-protein coupled receptor (GPCR) or G-protein regulator (GPR), dissociate and independently activate signaling cascades [27,28,29]. Upon ligand binding, the GPCR acts as a guanine nucleotide exchange factor (GEF), exchanging GDP for GTP on the α subunit, and activating the heterotrimeric G-protein. Heterotrimeric G-proteins can also be activated via a receptor-independent mechanism facilitated by G-protein regulator proteins (GPRs) (Figure 2) [30,31].

Upon activation, the GTP-bound α subunit disassociates from the βγ subunit, and both the α and βγ subunits can initiate downstream signaling pathways [32]. *C. elegans* expresses 21 Gα subunits [27] of the Gs, Gi/o, Gq, and G12 families, including only one ortholog of Gα_s_ (GSA-1) and Gα_i/o_ (GOA-1) [33]; these Gα subunits are typically upstream of adenylyl cyclase. *C. elegans* express two Gβ subunits, GPB-1 and GPB-2 and two γ subunits, GPC-1 and GPC-2. GPB-1 shares 86% homology with mammalian β subunits and interacts with all Gα subunits in *C. elegans* [34,35]. GPC-1/γ is expressed in sensory neurons, while GPC-2/γ is expressed more broadly [36]. Gβγ subunits can regulate ion channels [37,38] including Ca^2+^ channels [39], as well as activate or inhibit adenylyl cyclase [40]. GPB-2 acts downstream of the Gα subunit GOA-1 in pharyngeal pumping [41], and is required for egg-laying and locomotion [42,43], and works with GPB-1 and GSA-1 to regulate Ca^2+^ signaling and contractility in the spermatheca [44].

## 5. Ca^2+^ and cAMP Signaling Are Intertwined

The second messenger, Ca^2+^, is implicated in a variety of essential biological processes [45], making it critical to ensure correct concentration and localization. To maintain low Ca^2+^ concentrations in the cell, Ca^2+^ is pushed into the endoplasmic reticulum (ER) by SERCA pumps [46] or out of the cell by plasma membrane Ca^2+^ ATPases [47]. Gap junctions can mediate Ca^2+^ signaling between cells [48]. Channels located at the plasma membrane (PM), such as voltage-operated Ca^2+^ (VOCCs), receptor-operated Ca^2+^ channels (ROCCs), mechanically activated Ca^2+^ channels, transient receptor potential (TRP) ion channels, and store-operated Ca^2+^ channels (SOCCs) [49], also regulate the supply of Ca^2+^ from the extracellular space, and Ca^2+^ can be sequestered by the mitochondria [50]. Activation of phospholipase C (PLC) leads to the hydrolysis of phosphatidylinositol 4,5-bisphosphate (PIP_2_) to produce inositol 1,4,5-trisphosphate (PIP_3_) and diacyl glycerol (DAG). PIP_3_ binds to the IP_3_ receptor (IP_3_R) on the ER to release Ca^2+^. G-proteins mediate this release primarily through phospholipase PLCβ [51].

Numerous studies describe the complex and intertwined relationship of PKA, cAMP, and Ca^2+^ signaling [52]. For example, PKA can activate the ITR-1/IP_3_ receptor [53] and the ryanodine receptors (RYR), which mediate Ca^2+^ release from the ER in muscle and some non-muscle cell types [54,55]. PKA can regulate Ca^2+^ release by activating plasma membrane channels, such as stretch-sensitive TRPV channels [56]. In mouse cardiomyocytes, Gα activation can stimulate Ca^2+^ release through exchange protein directly activated by cAMP (EPAC) and Rap1 by stimulating PLCε [57,58]. However, PKA does not always stimulate Ca^2+^ release; in rat brain cells, phosphorylation of IP_3_R by PKA-C decreases Ca^2+^ release [59]. PKA can lower cytosolic Ca^2+^ levels by increasing the activity of SERCA pumps, which pump Ca^2+^ back into the ER, by phosphorylating and dissociating phospholamban [60,61]. PKA can inhibit PLC-β [62], which would result in decreased Ca^2+^ release. Additionally, adenylyl cyclases can be regulated by Ca^2+^ signaling [63], and IP_3_ receptors can be regulated by cAMP [64].

GPCRs regulate Ca^2+^ release through PKA in the *C. elegans* intestine. KIN-1/PKA-C plays a role in the *C. elegans* defecation cycle, which occurs rhythmically every 50 s [65]. The posterior, anterior, and enteric muscles contract sequentially to release waste [66]. GABAergic neurons (AVL and DVB) mediate the release of the neurotransmitter GABA, which prompts the release of gut contents by triggering muscle contractions [66,67,68,69]. KIN-1/PKA-C functions in the GABAergic neurons to regulate this expulsion step, acting downstream of the GPCR, AEX-2, and neuropeptide NLP-40. Constitutively active PKA in GABAergic neurons was sufficient to partially bypass loss of AEX-2, and PKA modulates muscle contraction and promotes Ca^2+^ influx into the DVB neurons through the voltage-gated Ca^2+^ channels UNC-2 and EGL-19 [65]. KIN-1/PKA-C stimulates the release of Ca^2+^ in neurons through specific voltage-gated calcium channels to control rhythmic defecation cycles.

Ca^2+^ and PKA signaling also coordinately regulate contractility in the *C. elegans* spermatheca, a smooth muscle-like tissue in the reproductive system and the site of fertilization in *C. elegans.* Cell contractility in the spermatheca is dependent on actin and myosin and is regulated, in part, by Ca^2+^ signaling through the phospholipase PLC-1 [70], which mediates Ca^2+^ release from the endoplasmic reticulum. GSA-1/Gα_s_, KIN-1/PKA-C, and KIN-2/PKA-R, regulate Ca^2+^ release and contractility in the *C. elegans* spermatheca [44]. Without GSA-1/Gα_s_ or KIN-1/PKA-C, Ca^2+^ is not released, and oocytes become trapped in the spermatheca. Conversely, when PKA is activated through either a gain of function allele in GSA-1 or by depletion of KIN-2/PKA-R, Ca^2+^ pulses continuously propagate across the spermatheca, even in the absence of oocyte entry. In the spermathecal–uterine valve, which connects the spermatheca to the uterus, loss of GSA-1/Gα_s_ or KIN-1/PKA-C results in the opposite phenotype: sustained, high levels of Ca^2+^ and a loss of coordination between the spermathecal bag and sp-ut valve. The phospholipase PLC-1 is required for these Ca^2+^ pulses. These results suggest activation of PKA has tissue-specific effects on the timing and intensity of Ca^2+^ release, and that KIN-1/PKA-C stimulates Ca^2+^ release downstream of GSA-1 in a PLC-1-dependent manner in the *C. elegans* spermatheca.

cAMP and Ca^2+^ signaling are also involved in neuronal regeneration in *C. elegans*. *C. elegans* neurons regenerate after laser axotomy [71,72]. PLM sensory neuron axotomy triggers a Ca^2+^ transient, which correlates with regenerative growth in late larval (L4) stage *C. elegans.* Genetically increasing Ca^2+^ or cAMP accelerates this growth, facilitates fusion of axonal fragments, and promotes branching; while inhibiting Ca^2+^ release reduces regrowth [73]. Inhibition of the regulatory subunit, through a loss of function allele *kin-2(ce179)* allele, promoted regeneration regrowth and elevated rates of fusion, while use of the PKA inhibitor H89 resulted in reduced regrowth in a dose-dependent manner [73]. In the ASJ neuron, cAMP signaling elevation or Ca^2+^ channel disruption improve DLK-independent regeneration [74]. Therefore, elevation of cAMP promotes neuronal regeneration in a PKA-dependent manner.

## 6. Oocyte Maturation

In *C. elegans,* major sperm proteins (MSP), which also polymerize to drive sperm motility, are released from sperm and stimulate oocyte meiotic maturation and oocyte production [75,76,77]. MSP and Gα-adenylyl cyclase signaling is required in the gonadal sheath cells and in the oocytes to regulate oocyte growth and meiotic maturation, possibly by antagonizing gap-junction communication between the sheath cells and oocytes [78]. Activating Gα-adenylyl cyclase signaling is sufficient to drive oocyte meiotic maturation in the absence of sperm [78,79]. KIN-1/PKA is required for oocyte meiotic maturation and functions downstream of ACY-4/adenylyl cyclase [80,81]. SACY-1, a highly conserved DEAD-box helicase that functions downstream of PKA, a two-pore domain potassium (TWIK) channel, and multiple components of a CoREST-like complex suppress *acy-4(lf)* sterility [80], suggesting they act downstream of Gα_s_–ACY-4–PKA to regulate oocyte meiotic maturation.

## 7. PKA Regulates Lipid Metabolism

In mammalian thermogenesis, β3-adrenergic receptor stimulation of G-proteins leads to the production of cAMP, and, therefore, PKA activation [82]. PKA activates hormone-sensitive lipase (HSL), which releases the glycerol and fatty acids required for the physiological activation of uncoupling protein 1 (UCP1), leading to increased production of heat [83]. Although *C. elegans* do not regulate body temperature [84], KIN-1/PKA-C does regulate *C. elegans* response to cold stress [85]. Under cold conditions, PKA signaling is activated in the intestine, where KIN-1 activity leads to increased expression of *hosl-1*/HSL, fat hydrolysis, increased glycerol availability, and increased cold tolerance. KIN-1 is also required in the neurons for cold tolerance. Although the mechanism is not known, perhaps neurons pass a signal to the intestine, which then upregulates *hosl-1* expression and lipid hydrolysis [85].

**Figure 3:**
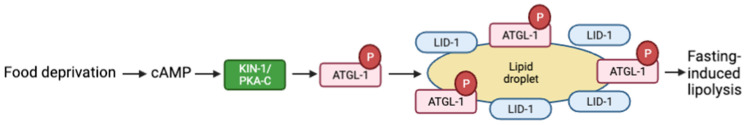
**Schematic representation of fasting-induced lipolysis model via the cAMP pathway**. Food deprivation increases cAMP and activates PKA. PKA-C activates the adipose triglyceride lipase ATGL-1, which then forms a liquid droplet with lipid droplet protein LID-1, which leads to lipid hydrolysis. Adapted with permission from [86], 2022, American Society for Microbiology. Created with BioRender.com (accessed on 23 October 2022).

PKA activation also results in lipolysis of stored lipid droplets in response to food deprivation. KIN-1 phosphorylates and stimulates the adipose triglyceride lipase ATGL-1 to form a lipid droplet-localized protein complex containing ATGL-1 and the lipid droplet protein LID-1, leading to lipid hydrolysis. The suppression of *atgl-1* or *lid-1* hinders fasting-induced lipolysis in adult worms (Figure 3) [86]. Lipolysis is reduced in low-oxygen conditions. Hypoxia-inducible factor (HIF) is a transcription factor that drives adaptive responses to low-oxygen levels, including the suppression of lipolysis. In mammals and *C. elegans*, hypoxia reduces cAMP and PKA activity levels, although the mechanism by which HIF-1 regulates PKA signaling in *C. elegans* is not clear. Exposure of *C. elegans* to hypoxia (1% O_2_) prevents PKA-stimulated lipolysis by targeting ATGL-1 for proteasomal degradation [87]. These studies are examples of the interaction between PKA activation and core metabolic processes.

PKA regulation of lipid metabolism impacts overall lifespan in most organisms [88,89,90]. In *C. elegans*, PKA activation of lipid catabolism in muscle cells and subsequent induction of AMPK/AAK-2 expression in non-muscle tissues, including neurons and intestinal cells, leads to enhanced mitochondrial metabolism and lifespan extension. Activating KIN-1/PKA-C and ATGL-1 in muscle cells led to a decrease in abundance of intramyocellular lipid and an extension of lifespan. Conversely, depletion of *atgl-1* in all tissues shortened lifespan [91].

## 8. PKA Regulates Mitochondrial Function and Lifespan

Mitochondrial fusion and fission are important for mitochondrial network morphology, biogenesis, embryonic development, metabolism, and apoptosis. Upon hypoxia or ischemia (low oxygen and glucose-deprived conditions), decreased availability of A-kinase anchoring protein 121 (AKAP121) by Siah2 (seven in absentia homolog (SIAH) family) leads to mitochondrial fission and cell death in mice [92]. Dynamin-related protein 1 (Drp1), a direct target of PKA, encodes a dynamin-like GTPase that controls mitochondrial fission [93]. AKAP121 binding of PKA blocks phosphorylation of Drp1, which prevents the formation of a complex between Drp1 and the outer mitochondrial membrane protein Fis1 (fission, mitochondrial 1) and subsequent mitochondrial fission. In *C. elegans*, inhibition of *siah-1* or *drp-1* during larval development shortens lifespan [92], presumably through effects on mitochondrial activity or dynamics.

PKA is required for *C. elegans* lifespan extension in a variety of contexts. For example, *C. elegans* with defective oxidative phosphorylation due to a mutation in the respiratory complex I subunit, GAS-1, are short lived. Inhibition of insulin/IGF signaling in these animals through loss of DAF-2/IGF1 receptor or AGE-1/PI3K rewires the animal’s metabolism and extends lifespan [94]. This effect depends on PKA signaling; *kin-1(RNAi)* abrogates the lifespan extension of *age-1*; *gas-1* double mutants. The purine base xanthine increases when insulin signaling is inhibited. Treatment of animals with xanthine derivatives increases AAK-2/AMPK and KIN-1/PKA activity, enhances mitochondrial network remodeling, and induces the metabolic changes that give rise to the lifespan extension [95]. In addition, DAF-2/IGF1 receptor and AGE-1/PI3K inhibit KIN-1/PKA-C in dauer-stage animals [96].

In mammalian cells and *C. elegans*, PKA phosphorylates and activates SIRT1, a sirtuin protein, which leads to improved mitochondrial function and fatty acid oxidation. Hydralazine, a drug used for hypertension, heart failure, and cancer treatment, improves mitochondrial function and elevates SIRT1 levels in cells [97]. In *C. elegans*, hydralazine extends lifespan through a PKA-dependent mechanism. Hydralazine binds to the catalytic subunit KIN-1/PKA-C, enabling separation from the regulatory subunit and activating PKA. PKA activation contributes to both SIRT1 activation and to the stress regulatory SKN-1/NRF2 signaling pathway, resulting in increased lifespan through glucose-induced mitochondrial dysfunction. Further studies are required to discover the mechanism by which PKA regulates SIRT1 and NRF2 [97].

PKA is part of a signaling pathway that regulates nucleotide metabolism and reproductive development in response to nucleotide imbalance in the gut of *C. elegans*. During genotoxic stress, Nucleotide (NT) deficiency stimulates the nucleotide-sensing system that mediates mitotic germline proliferation and NT metabolism in the intestine. The poly(U)-specific endoribonuclease, ENDU-2, is a regulator that reacts to NT imbalance and genotoxic stresses. ENDU-2 regulates of CTPS-1, a cytidine triphosphate (CTP) synthase, by both inhibiting KIN-1/PKA-C signaling, possibly by repressing adenylyl cyclase activity, and by regulating histone deacetylase HDA-1 activity. This prevents activation of the cytidine triphosphate (CTP) synthase CTPS-1, which inhibits proliferation under genotoxic stress and increases lifespan [98]. Although these studies suggest an important role for PKA, many questions remain regarding the mechanisms by which PKA regulates lifespan extension, mitochondrial dynamics and metabolism.

## 9. PKA Signaling in Neurons Regulates *C. elegans* Behaviors

**Figure 4:**
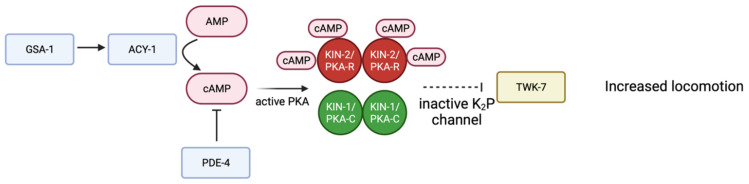
**Schematic representation of proposed signaling pathway in B- and D- type motor neurons**. G_αs_ GSA-1 activates adenylyl cyclase ACY-1, producing more cAMP and therefore activating PKA. TWK-7 is a K_2_P channel that normally decreases locomotion. Through a mechanism that is not entirely understood (dotted line) active PKA inhibits TWK-7, therefore increasing locomotion. Adapted with permission from [99], 2022, Oxford University Press. Created with BioRender.com (accessed on 23 October 2022).

Several studies suggest PKA activity in neurons regulates locomotion. Activation of KIN-1/PKA-C, through depletion of *kin-2*, results in hyperactive movement of *C. elegans*; similar phenotypes are seen when the Gα_s_ protein GSA-1 is activated or the phosphodiesterase PDE-4 is depleted [99]. Loss of function alleles in the two-pore-domain potassium (K_2_P) channel TWK-7 increase locomotion. Genetic evidence suggests TWK-7 is downstream of the GSA-1-KIN-1/PKA pathway in B- and D-type motor neurons [99]. Activation of KIN-1/PKA-C inhibits TWK-7. When PKA is active, TWK-7 is repressed, leading to increased locomotion. Further studies are necessary to understand the mechanism by which KIN-1/PKA-C regulates TWK-7 in motor neurons (Figure 4) [99].

The neurotransmitter acetylcholine stimulates *C. elegans* locomotion. Several studies have revealed a role for PKA in acetylcholine release in motor neurons [100]. Exposure of *C. elegans* to 0.1% ethanol also increases locomotion. Although the precise mechanism by which ethanol increases locomotion remains unclear, the Gαs-cAMP-PKA signaling pathway can be activated by ethanol in the IL2 sensory neurons, which release acetylcholine and link to locomotor circuits by intermediary neurons [101]. This study identified a key downstream effector of PKA signaling, UNC-18/Sec1-Munc18. UNC-18 plays an essential role in synaptic vesicle exocytosis. Gαs signaling activates KIN-1/PKA-C, which phosphorylates UNC-18 thereby increasing neurotransmitter release and stimulating *C. elegans* locomotion [101]. A similar pathway may be involved in the response to isoflurane, a general anesthetic, which results in erratic and diminished neuronal activity in motor neurons and physical quiescence of the nematode [102]. Sensitivity to isoflurane is related to levels of acetylcholine release. Activating KIN-1/PKA-C through loss of *kin-2* or a gain of function mutation in adenylyl cyclase *acy-1(js127)* results in resistance to isoflurane [103]. Aldicarb treatment increases acetylcholine release and leads to sustained muscle activation and eventual paralysis. Activation of KIN-1 further increases acetylcholine release and increases sensitivity to aldicarb-induced paralysis [104].

In addition to locomotion, PKA signaling in neurons regulates other behaviors, including wakefulness. In *C. elegans*, Drosophila, and mice, increased PKA-1 activity promotes wakefulness [105,106,107,108], via the transcriptional activator cAMP response element-binding protein (CREB) [108]. When KIN-1/PKA-C activity is increased by deletion of KIN-2/PKA-R or ACY-1(GF), the worms are more active. PKA-C activates the transcription factor CRH-1/CREB and promotes neuropeptide release to promote active wakefulness. CRH-1 is the CREB ortholog in *C. elegans* [105,109]. The Ca^2+^-dependent activator protein for secretion UNC-31/CAPS is necessary for neuropeptide release from dense core vesicles (DCV). By enhancing mobilization and priming, cAMP/PKA signaling augments synaptic vesicle (SV) fusion [110]. Activation of PKA can bypass the requirement for UNC-31 in the docking of DCVs in exocytosis. KIN-1/PKA-C phosphorylates the syntaxin-1-binding protein, TOM-1, which downregulates synaptic transmission and UNC-31/CAPS-dependent neuropeptide release, resulting in locomotion regulation and stabilized wakefulness [105,111].

CREB is a common downstream effector of PKA. PKA activation of CRH-1/CREB also regulates the level of the FMRFamide-related neuropeptide FLP-19 in BAG sensory neurons, contributing to CO_2_ sensing and response [112]. PKA signaling through CREB also enhances neural circuit excitability and improves memory. *Eleutheroside E*, a sterol glycoside extracted from Siberian ginseng, *Eleutherococcus senticosus* [113], has a neuromodulatory effect and protects radiation-damaged nerves. This compound signals through Gαq and PLC to activate cAMP-PKA, improving performance on associative learning assay and memory tasks. Through downstream activation of the transcriptional regulator CREB and expression of neuropeptides, *Eleutheroside E* increases long-term memory of radiation-damaged *C. elegans* in AIM and AWC neurons, respectively [114].

**Figure 5:**
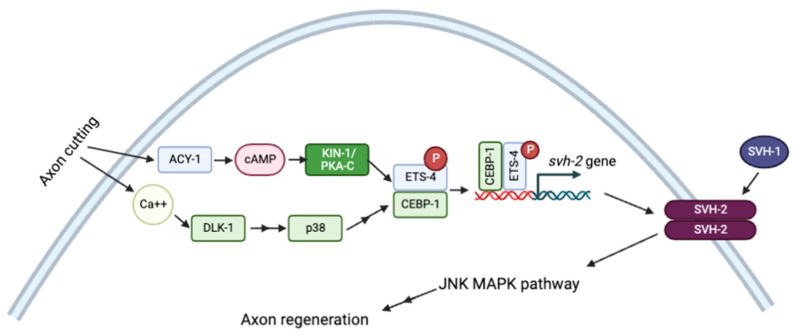
**Schematic representation of axon regeneration model through JNK MAPK pathway by Ca^2+^ and cAMP signaling pathways.** Adapted with permission from [115], 2022, Creative Commons. Created with BioRender.com (accessed on 23 October 2022).

Signaling through cAMP/PKA also modulates axonal regeneration in many systems. In response to injury, cAMP/PKA-dependent phosphorylation activates the transcription factor ETS-4, which interacts with CEBP-1 to upregulate the expression of the receptor tyrosine kinase SHV-1. Activation of *svh-2* expression requires simultaneous Ca^2+^ signaling and activation of the p38 MAPK pathway. SVH-2 then activates the JNK MAPK pathway, which stimulates axon regeneration (Figure 5) [115].

## 10. PKA Action in Neurons Regulates *C. elegans* Physiology

In addition to the effects on lifespan, movement, and behavior, PKA action in neurons regulate a wide variety of different processes in *C. elegans*. For example, the neuromodulator serotonin (5-hydroxytryptamine, 5-HT), released by maternal neurons upon stress, can activate the transcription factor heat shock factor 1 (HSF-1) through PKA signaling in the germline, mediating the histone chaperone FACT (facilitates chromatin transcription) and promoting viability and future stress tolerance. For example, embryos produced from the heat-shocked mothers have more protective mRNA and are better able to tolerate high temperatures as larvae. [116].

In vertebrates, melatonin, which influences circadian rhythms, is produced by arylalkylamine N-acetyltransferase (AA-NAT) and N-acetylserotonin methyltransferase (ASMT) [117]. The AA-NATs are broadly expressed, including in many neurons. Light inhibits AA-NAT activity, allowing for a day/night rhythm. In dark–light conditions, *C. elegans* also produces a rhythmic pattern of melatonin levels [118]. Nine putative *C. elegans* AA-NATs were found with PKA phosphorylation sites [118], providing a possible mechanism by which PKA could regulate circadian rhythms.

KIN-29 is a serine/threonine kinase of the SIK (salt-inducible kinase) family that regulates chemoreceptor gene expression by phosphorylating and inhibiting histone deacetylases [119]. cAMP is produced in the CAN (canal-associated neurons) and dissociates from the CANs through gap junctions to the target cells to regulate PKA and KIN-29, which in turn, regulates larval development. When KIN-29 is present, larval development is inhibited. PKA inhibition of KIN-29 is necessary for larval development to proceed [120].

## 11. Immunity

The KIN-1/PKA-C pathway is critical for *C. elegans* immune response to infection by *S. enterica, P. aeruginosa,* and *S. aureus*. The adenylyl cyclase ACY-1 regulates the innate immune response to pathogens through activation of KIN-1/PKA-C [121]. Neuronal-specific knockdown of *kin-1* by RNAi contributes to a decline in the survival rate of WT worms infected with *S. enterica* and inhibition in the expression of some antimicrobial and lysosomal genes. KIN-1 upregulates antimicrobial genes including lysozymes, caenopores, C-type lectins, caenacins, and genes of the pqn family, among other factors. The lysosomal pathway mediates the downstream effects of PKA/KIN-1 signaling and controls autophagic flux and the lysosomal degradation rate. KIN-1/PKA-C action in the nervous system is critical for innate immunity, perhaps via release of an unknown signal that triggers these pathways in the intestine and epidermis [121].

## 12. Conclusions

PKA is a pleiotropic cellular regulator that wields powerful effects on diverse biological processes. Much has been done to elucidate the role of PKA in *C. elegans*. PKA plays vital roles in fertility, lipid metabolism, mitochondrial function and lifespan, and *C. elegans* behaviors and physiology. Because PKA activity is required for such a broad set of roles, PKA activity is tightly controlled. Several different mechanisms contribute to specificity in PKA signaling, including expression of specific isoforms, protein modifications that affect intracellular targeting, and binding of PKA-R subunits to A kinase-anchoring proteins (AKAPs), which control signaling output by enabling association with specific effectors, facilitating spatial and temporal compartmentalization of PKA signaling.

The relative simplicity and genetic tractability of the *C. elegans* offers an opportunity for further discovery of novel regulators and effectors of PKA signaling. For example, the large, polarized, and easily visible cells of the spermatheca offer an opportunity to observe KIN-2/PKA-R localization during ovulation and oocyte transit, and to assess the dependency of this localization on AKAP-1, ERM-1, and/or yet to be identified factors that may function as AKAPs in *C. elegans*. In addition, the *C. elegans* somatic gonad, comprised of gonadal sheath cells, spermathecal cells, sp-ut valve and uterus, offers an excellent system for genetic screens that will improve our understanding how PKA regulates coordinated Ca^2+^ signaling between and among cell types in a tissue. Of particular interest is how Ca^2+^ release is inhibited by PKA in some cell types and stimulated in others. As in previous work, paradigms identified in *C. elegans* may apply across organisms in this well conserved signaling pathway.

## Data Availability

Not applicable.
